# Fluid Mechanical and Visible-Light-Driven Piezophotocatalysis in MoS_2_/Carbon-Rich Carbon Nitride Heterostructures for Enhanced Green Energy Production and Environmental Remediation

**DOI:** 10.1021/acsami.5c01107

**Published:** 2025-03-01

**Authors:** Chien-Jung Wu, Sin-Cin He, Tzu-Chi Kuo, Jih-Jen Wu

**Affiliations:** Department of Chemical Engineering, National Cheng Kung University, Tainan 701, Taiwan

**Keywords:** molybdenum disulfide, carbon-rich carbon nitride, fluid flow, visible light, piezophotocatalysis

## Abstract

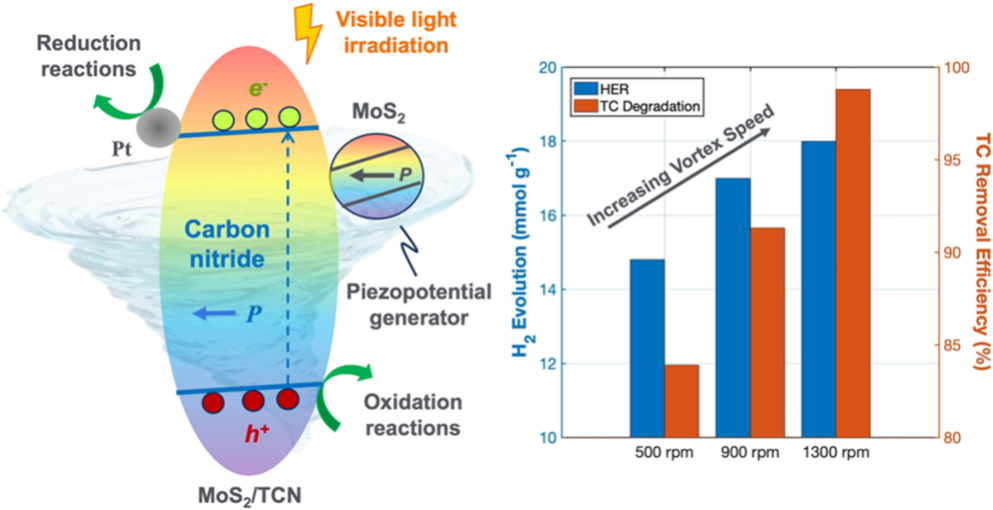

Molybdenum disulfide (MoS_2_)/carbon-rich carbon nitride (TCN) heterostructure, a piezophotocatalyst sensitive to fluid mechanical energy and visible light, has been developed for green energy production and environmental remediation. The optimized MoS_2_/TCN heterostructure exhibits an absorption edge at 520 nm, identical to that of TCN but significantly red-shifted compared with conventional carbon nitride. Piezopotential measurements via piezoelectric force microscopy demonstrate that the MoS_2_/TCN heterostructure generates a much higher piezopotential response than TCN under the same applied voltage. This heterostructure exhibits substantial improvements in photocatalytic performance for both the hydrogen evolution reaction (HER) and the degradation of tetracycline (TC) under visible light. Additionally, its photocatalytic activity is further enhanced by vortex-induced fluid motion. Compared to TCN, the piezophotocatalytic activity of the optimized MoS_2_/TCN heterostructure increases the HER rate from 1.8 to 3.62 mmol g^–1^ h^–1^ and the TC degradation rates from 57.8 to 85.1% and 73.2 to 98.8% in 15 and 60 min, respectively. MoS_2_ nanosheets act as piezoelectric generators, triggered by fluid flow, to induce a macroscopic piezopotential, aiding in the collection of visible-light-generated electrons and holes on the TCN surface to enhance catalytic activity. This work highlights that the shearing forces from fluid flow, essential for wastewater discharge, piezoelectrically amplify the photocatalytic efficiency of the MoS_2_/TCN heterostructure.

## 1 Introduction

“Affordable and clean energy” and “clean water and sanitation” are two of the 17 goals highlighted in Sustainable Development Goals Report 2024 by the United Nations.^1^ The demand for energy has increased significantly in recent years due to population growth and rising living standards.^2,3^ Excessive fossil fuel consumption has contributed to the worsening of global warming and the degradation of air quality.^4−6^ To address the rise in greenhouse gas emissions and promote renewable energy use, various approaches, including carbon dioxide reduction,^7^ nitrogen fixation,^8,9^ and hydrogen evolution,^10,11^ are being pursued. Novel catalysts for clean energy production are being developed to achieve net-zero carbon emissions by 2050.^12^ Hydrogen is recognized as a sustainable and eco-friendly energy source, providing significant advantages, including a high energy density of up to 140 MJ kg^–1^ and zero carbon emissions.^13^ Photocatalytic water reduction by renewable solar energy through semiconductors is a prospective strategy for green hydrogen production with no reliance on fossil fuel and no carbon dioxide emission.^14^

In addition, resolving degradation in water quality and severe freshwater scarcity is essential to achieve the goal of clean water and sanitation, which is becoming increasingly challenging and requires innovative technology.^15,16^ Antibiotics such as tetracycline (TC) are widely used as growth promoters in livestock and as antibacterial medications for humans. Previous studies have shown that TC is present downstream of wastewater treatment plants at concentrations similar to those in the effluent.^17^ These antibiotics are significant pollutants in natural aqueous environments globally due to increased antibiotic abuse from aggressive pharmaceutical demand. Antibiotics in aqueous environments not only build up in aquatic organisms but also promote the development of antibiotic-resistant bacteria in the environment.^18,19^ Antibiotics can be removed from wastewater through various treatment methods, including membrane technology,^20,21^ biological methods,^22,23^ and photocatalytic reactions.^24,25^ Among them, the photocatalytic technique possesses the advantages of environmental friendliness and low cost, suitable for antibiotic degradation.^18^ As a metal-free semiconductor, carbon nitride has recently attracted considerable attention for its application as a photocatalyst for green hydrogen production and environmental remediation.^26^ Various modification strategies, such as constructing heterostructure^27^ and introducing defects,^28^ have been demonstrated to further improve the photocatalytic performances of carbon nitrides.

Piezophotocatalysis, coupling the piezoelectric effect with the light-absorbing ability of semiconductor catalyst, has recently emerged as a potential process for hydrogen evolution reaction (HER) and degradation of the organic pollutants in water.^29−33^ A macroscopic potential field is built in the piezophotocatalyst responding to applied stress. This facilitates the separation and transport of the photogenerated charges in the catalyst to enhance catalytic reactions.^34,35^ Ultrasonic waves are commonly used to demonstrate piezophotocatalytic processes;^36,37^ however, the lack of natural sources of ultrasonic energy^38^ and the influence of sonochemical effects^39^ raise concerns about the feasibility of this approach for real-world applications. To sustainably implement piezophotocatalytic technology for HER and the degradation of organic pollutants in water, it is essential to combine solar energy with fluid mechanical energies available in nature. Specifically, piezophotocatalytic degradation of organic pollutants can be efficiently conducted during wastewater discharge, as the piezopotential in the catalyst is drivable by fluid mechanical energy. Accordingly, the development of fluid mechanical energy-sensitive piezophotocatalysts for clean energy production and wastewater treatment is a potential strategy for achieving the goals of “affordable and clean energy” and “clean water and sanitation”.

Previous work has shown that two-dimensional (2D)-2D-stacked molybdenum disulfide (MoS_2_)/carbon nitride exhibits enhanced piezophotocatalytic HER activity when activated by vortex flow.^40^ However, the carbon nitride matrix in this piezophotocatalyst is limited in its light-harvesting capability to wavelengths below 460 nm. Substituting nitrogen sites in the aromatic rings with carbon atoms has been reported to improve charge transport and extend optical absorption to longer wavelengths, thereby enhancing photocatalytic activity.^41^ Additionally, carbon nitride-based photocatalysts have demonstrated promising results for antibiotic degradation in water.^42,43^ However, piezophotocatalytic antibiotic degradation driven by fluid flow and visible light over carbon nitride-based catalysts has not yet been reported. In this study, MoS_2_/carbon-rich carbon nitride heterostructures, responsive to both visible light and fluid mechanical energy, were developed by coupling the visible-light photocatalytic properties of carbon-rich carbon nitride^41^ with the piezoelectric characteristics of MoS_2_^44^ for piezophotocatalytic HER and tetracycline (TC) degradation. This heterostructure shows substantial improvements in photocatalytic performance for both HER and TC degradation under visible light combined with vortex-induced fluid motion. The role of the MoS_2_ nanosheets in piezophotocatalytic reactions is also investigated to elucidate how fluid flow-induced shearing forces enhance the photocatalytic efficiency of the MoS_2_/TCN heterostructure through piezoelectric effects.

## 2 Experimental Section

### 2.1 Synthesis of Molybdenum Disulfide-Carbon-Rich Carbon Nitride (MoS_2_/TCN) Heterostructures

Carbon-rich carbon nitride (TCN) was prepared through a supramolecular self-assembly process followed by thermal condensation, as reported in the previous work.^41^ In brief, the aqueous solution of melamine (MA), cyanuric acid (CA), and 2,4,6-triaminopyrimidine (TAP) with a molar ratio of 0.97:1:0.03 was vigorously stirred for 24 h at room temperature. After the precursor solution was filtered, the retentate was dried in a vacuum oven overnight to have the supramolecular complex. TCN was obtained from the calcination of the supramolecular complex at 600 °C for 4 h with a ramping rate of 10 °C min^–1^.

MoS_2_/TCN heterostructures were prepared by the solvothermal deposition of MoS_2_ nanosheets on TCN. 3 mg of ammonium tetrathiomolybdate was dispersed in 20 mL of dimethylformamide (DMF) and was subjected to an ultrasonic treatment for 30 min to form the MoS_2_ precursor solution. Then, 20 mg of TCN was dispersed in 25–*x* mL of DMF under magnetic stirring for 30 min, followed by introducing *x* mL of the MoS_2_ precursor solution (*x* = 1, 10, and 20 mL) and further stirring the mixture for another 30 min. The mixed solution was transferred into a Teflon-lined stainless-steel autoclave and was maintained at 200 °C for 15 h. For simplicity, the MoS_2_/TCN heterostructures constructed using 1, 10, and 20 mL MoS_2_ precursor solutions are named 1-MoS_2_/TCN, 10-MoS_2_/TCN, and 20-MoS_2_/TCN, respectively.

### 2.2 Characterizations of MoS_2_/TCN Heterostructures

The surface morphologies of TCN and MoS_2_/TCN heterostructures were inspected by high-resolution field-emission scanning electron microscopy (HR-FESEM, SU-8010, Hitachi) under an accelerating voltage of 10 kV. The elemental distributions of TCN and MoS_2_/TCN were examined using energy-dispersive spectroscopy (EDS, X-maxN, Hobria) equipped on the FESEM. The crystal structures were characterized by X-ray diffraction (XRD, D8 Discover, Bruker) and high-resolution transmission electron microscopy (HRTEM, JEM-2100F CS STEM, JEOL). X-ray photoelectron spectroscopy (XPS, K-α, Thermo Scientific) was used to evaluate the chemical states of TCN and the MoS_2_/TCN heterostructure. UV–vis diffuse reflectance spectra (DRS) were measured by a spectrophotometer (V-670, Jasco) equipped with a UV–visible/NIR integrating sphere. Time-resolved photoluminescence (TRPL) measurements were carried out by a pulsed 405 nm laser and a time-correlated single photon counting spectrometer. Piezoelectric responses of TCN and MoS_2_/TCN heterostructures were examined using piezoelectric force microscopy (Dimension ICON, Bruker). The drive voltage applied to the tip for the piezoelectric response measurements is 3 V. The deflection sensitivity is calibrated based on piton sapphire (107.3 nm V^–1^). All images were analyzed by using NanoScope software (Bruker).

### 2.3 Piezophotocatalytic Reactions over MoS_2_/TCN Heterostructures

The piezophotocatalytic HER was carried out in an anaerobic reactor under AM 1.5G (100 mA cm^–2^) irradiation (LCS-100, Newport) with an ultraviolet (UV) cutoff filter at 420 nm. A digital vortex mixer (Digital Vortex-Genie 2, Scientific Industries, Inc.) was utilized as the fluid mechanical force provider. 5 mg of catalyst with 1.0 wt % Pt was dispersed in 10 mL of aqueous solution with 10 vol % triethanolamine (TEOA). The H_2_ generation was inspected using a gas chromatography (GC, GC2014, Shimadzu) system with a thermal conductivity detector.

The piezophotocatalytic TC degradation was conducted in an aerobic condition under AM 1.5G (100 mA cm^–2^) irradiation with a UV cutoff filter at 420 nm coupling with vortex flow. 5 mg of catalyst was dispersed in a 10 mL TC solution (10 ppm). The mixture underwent ultrasonic treatment for 10 min to achieve a uniformly dispersed solution. The system was initially kept in the dark for 90 min to reach adsorption–desorption equilibrium. During a 60 min piezophotocatalytic reaction under visible-light irradiation and vortex flow, aliquots of the TC solution were extracted every 15 min for UV–vis absorption analysis. The characteristic absorption peak of TC at 357 nm was used to determine its concentration by converting the absorbance using a calibration curve. The catalysts used in the durability test were lyophilized after each reaction cycle and weighed to assess the degradation efficiency accurately. The sacrificial agent test was conducted under the same experimental conditions as those of the normal degradation reaction, with the only difference being the addition of varying amounts of sacrificial agents to the TC solution. Specifically, isopropyl alcohol (IPA) was added at a concentration of 10 mM, ethylenediaminetetraacetic acid disodium (EDTA-2Na) at 10 mM, and nitroblue tetrazolium chloride (NBT) at 0.015 mM.

### 2.4 Electrochemical Measurements

The electrochemical measurements were conducted by using a three-electrode photoelectrochemical cell with 0.5 M Na_2_SO_4_ supporting electrolyte. The catalyst electrode, a platinum wire, and an Ag/AgCl reference electrode were employed as the working electrode, counter electrode, and reference electrode, respectively. 1 mL of ethanol, 3 mg of catalyst, and 50 μL of Nafion (5.0 wt %) were vortex-mixed to form the catalyst suspension. To construct the catalyst electrode, 40 μL of catalyst suspension was dropped on the fluoride-doped tin oxide (FTO) glass twice, followed by dropping 40 μL of diluted Nafion solution (0.5 wt %) and staying under ambient conditions overnight. Electrochemical impedance spectroscopy (EIS) measurements were performed by applying a 10 mV AC in the frequency range of 10 mHz to 100 kHz at a direct current (DC) potential of −1.5 V vs Ag/AgCl using a potentiostat with a frequency response analyzer (Autolab PGSTAT 30 FRA2).

## 3 Results and Discussion

### 3.1 Characterizations of TCN and MoS_2_/TCN Heterostructures

**Figure 1:**
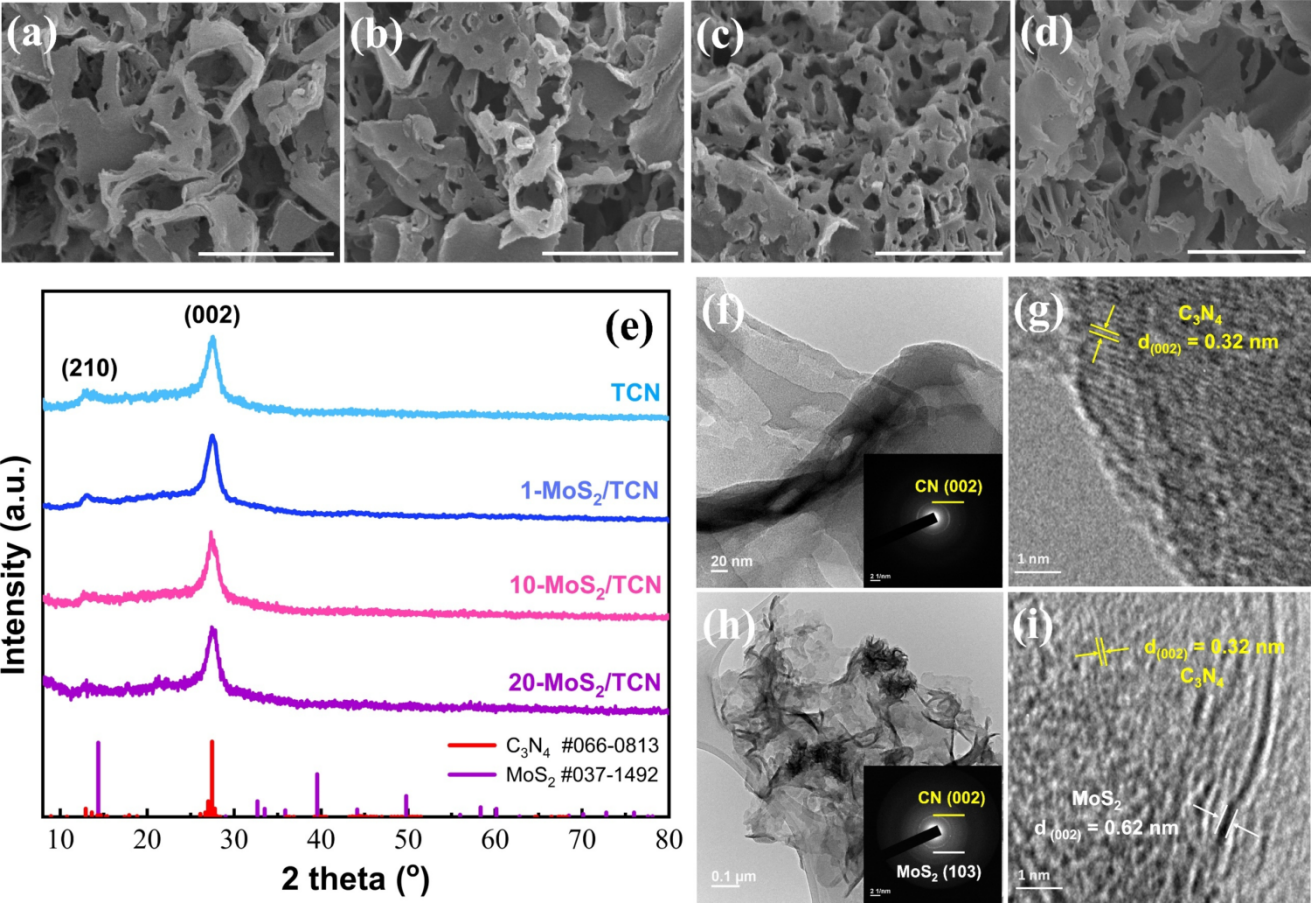
SEM images of (a) TCN, (b) 1-MoS_2_/TCN, (c) 10-MoS_2_/TCN, and (d) 20-MoS_2_/TCN (scale bar: 1 μm). (e) XRD patterns of TCN and MoS_2_/TCN heterostructures. (f) TEM image of TCN and the corresponding SAED pattern (inset). (g) HRTEM image of TCN. (h) TEM image of 10-MoS_2_/TCN and the corresponding SAED pattern (inset). (i) HRTEM image of 10-MoS_2_/TCN.

Figure 1a–d demonstrates the scanning electron microscopy (SEM) images of TCN, 1-MoS_2_/TCN, 10-MoS_2_/TCN, and 20-MoS_2_/TCN, respectively, revealing the porous sheet-like morphology of the catalysts. Compared with TCN, the MoS_2_/TCN heterostructures become more porous after the solvothermal MoS_2_ deposition process. SEM-EDS mapping images of TCN and 10-MoS_2_/TCN are shown in Figures S1 and S2, respectively. The EDS signals of S and Mo well distributed on the matrix composed of C and N elements are displayed in Figure S2. N_2_ sorption isotherms are acquired from TCN and 10-MoS_2_/TCN, as illustrated in Figure S3, to determine the specific surface area and pore volume distribution. The isotherm N_2_ sorption curves with shapes similar to a type IV isotherm indicate the mesoporous characteristic of both TCN and 10-MoS_2_/TCN. The Brunauer–Emmett–Teller (BET) surface areas of TCN and 10-MoS_2_/TCN are calculated to be 68.8 and 113.3 m^2^ g^–1^, respectively. A significant increase in the specific surface area of 10-MoS_2_/TCN is consistent with the observations from the SEM images. The enlarged average pore size from 17.1 nm of TCN to 25.2 nm of 10-MoS_2_/TCN also supports the finding.

The crystal structures of MoS_2_/TCN heterostructures were first examined by using XRD. The XRD patterns of TCN and MoS_2_/TCN heterostructures are shown in Figure 1e. The standard powder diffraction patterns of C_3_N_4_ (#066–0813) and MoS_2_ (#037–1492) are displayed in this figure for comparison. Two prominent peaks at 2θ = 13.1 and 27.6° present in the XRD patterns of TCN and MoS_2_/TCN heterostructures pertain to the (210) and (002) lattice planes of the melon carbon nitride,^45^ respectively. However, there is no diffraction peak of MoS_2_ detected from the MoS_2_/TCN heterostructures. The microstructures of TCN and 10-MoS_2_/TCN were further characterized using transmission electron microscopy (TEM) to validate the formation of the MoS_2_/TCN heterostructure. As shown in Figure 1f and its inset, the typical TEM image and the corresponding selected area electron diffraction (SAED) pattern of TCN composed of 2D curly sheets with the diffraction ring attributed to the (002) crystal plane of carbon nitride are inspected. The HRTEM image of TCN in Figure 1g exhibits clear lattice fringes with an interplanar spacing of 0.32 nm, which corresponds to the *d*-spacing of the (002) planes of carbon nitride. With MoS_2_ deposited on TCN, as shown in Figure 1h, a distinct contrast can be observed at the edges of the sheets in the TEM image of 10-MoS_2_/TCN, and the diffraction ring of MoS_2_ (103) appears in the corresponding SAED pattern. The HRTEM image in Figure 1i exhibits two sets of lattice fringes with spacings of 0.32 and 0.62 nm. They are assigned to the (002) planes of carbon nitride and MoS_2_, respectively, further confirming the successful construction of the MoS_2_/TCN heterostructure.

**Figure 2:**
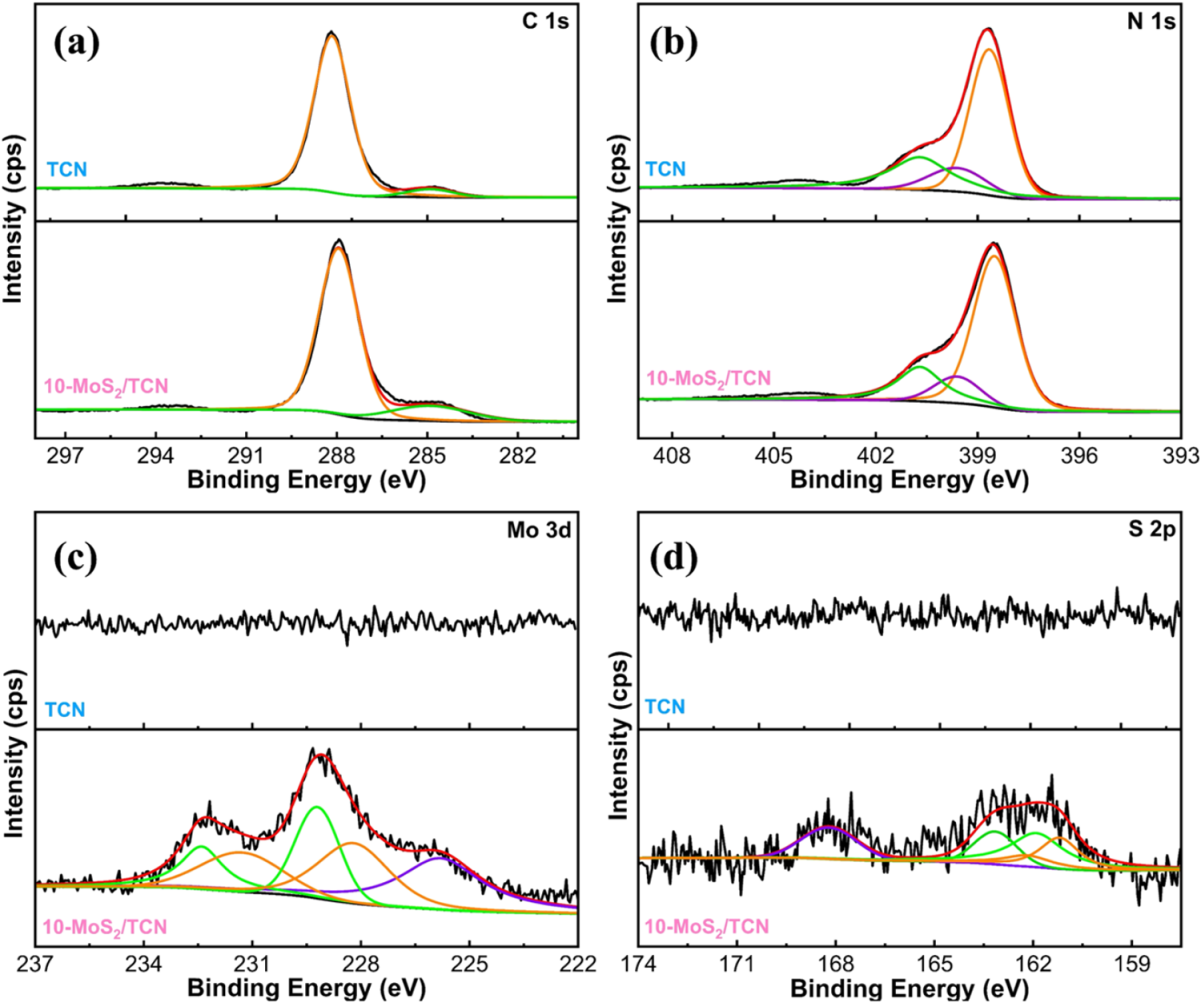
(a) C 1s, (b) N 1s, (c) Mo 3d, and (d) S 2p XPS spectra of TCN and 10-MoS_2_/TCN.

XPS spectra of TCN and 10-MoS_2_/TCN were analyzed to elucidate the chemical states of the MoS_2_/TCN heterostructures. Figure 2a shows the deconvoluted C 1s XPS spectra of TCN and 10-MoS_2_/TCN with 2 peaks at 288.5 and 284.9 eV, corresponding to sp^2^-hybridized carbon in aromatic rings (N–C=N) of carbon nitride and adventitious carbon, respectively.^46,47^ The N 1s spectra of TCN and 10-MoS_2_/TCN can be deconvoluted to 3 peaks at binding energies of 398.5, 399.6, and 400.7 eV, as illustrated in Figure 2b, pertaining to sp^2^-hybridized aromatic nitrogen (C=N–C), central nitrogen bonded with carbons (N–(C)_3_), and terminal amine groups (NH_*x*_), respectively.^46,47^ Based on the deconvolution results, the N atomic ratio of C=N–C:N–(C)_3_:NH_*x*_ for both TCN and 10-MoS_2_/TCN is estimated to be 5.8:1:1.8. This ratio is distinct from the theoretical value of 6:1:2 for melon carbon nitride,^48^ which is ascribed to the carbon-rich heptazine rings with the sp^2^ N partially substituted by the graphitic carbon atom and the terminal amino group partially replaced by the H atom in the TCN.^41^ Moreover, the N atomic ratio of 10-MoS_2_/TCN remains the same as that of TCN, suggesting that the chemical structure of carbon-rich carbon nitride is not altered after MoS_2_ deposition.

Figure 2c,d displays the Mo 3d and S 2p XPS spectra of TCN and 10-MoS_2_/TCN. No Mo and S signals are acquired from TCN, while noticeable signals appear in the spectra of 10-MoS_2_/TCN. Accordingly, XPS analysis also confirms the formation of MoS_2_/TCN heterostructure in 10-MoS_2_/TCN. The Mo 3d XPS spectrum of 10-MoS_2_/TCN in Figure 2c is deconvoluted to 5 peaks. The peak at the binding energy of 226.2 eV corresponds to S 2s.^49^ The other 4 peaks at 232.3, 231.4, 229.1, and 228.1 eV are ascribed to Mo 3d_3/2_ of 2H, Mo 3d_3/2_ of 1T, Mo 3d_5/2_ of 2H, and Mo 3d_5/2_ of 1T MoS_2_, respectively.^50^ Similarly, two sets of peaks located at 163.0, 161.9, 162.1, and 161.2 eV are deconvoluted from the S 2p spectrum of 10-MoS_2_/TCN in Figure 2d, corresponding to the S 2p_1/2_ and S 2p_3/2_ of both the 2H and 1T phases of MoS_2_.^50^ The peak located at around 168 eV is attributed to oxidized sulfur.^51^ Additionally, the Mo:S atomic ratio, estimated from the corresponding peak areas in Figure 2c,d, is 1:1.9, which closely aligns with the expected Mo:S ratio in MoS_2_.

**Figure 3:**
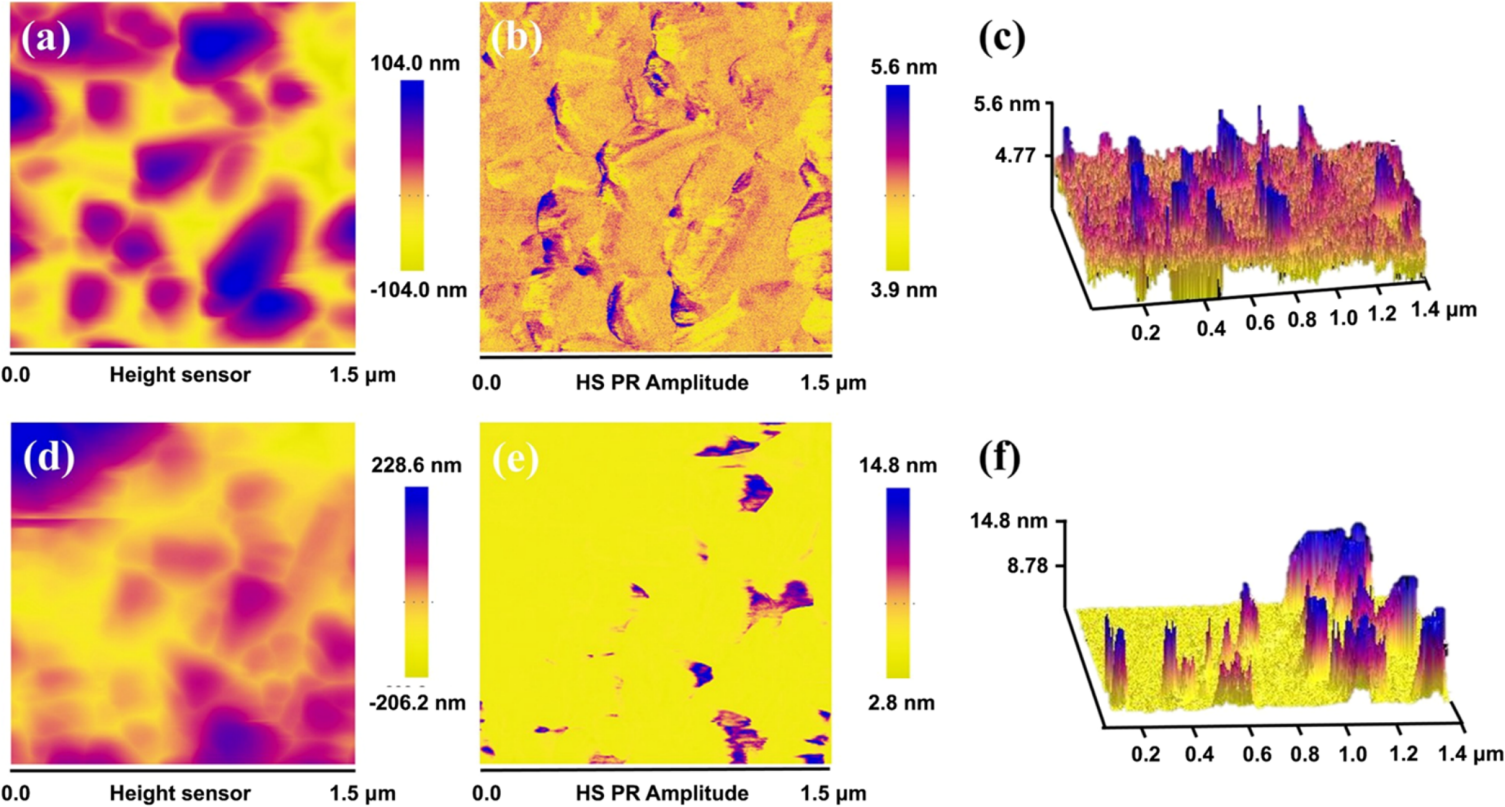
(a) 2D topological image. (b, c) 2D and 3D piezoelectric responses of TCN. (d) 2D topological image and (e, f) 2D and 3D piezoelectric responses of 10-MoS_2_/TCN.

The piezoelectric characteristics of TCN and 10-MoS_2_/TCN were examined using PFM. When the PFM tip imparts the electric field to the piezoelectric surface, the resultant strain in the *z*-direction will be recorded in amplitude and phase. Figure 3a shows the 2D topological graph of TCN. The corresponding 2D and three-dimensional (3D) piezoresponse amplitude images are depicted in Figure 3b,c, respectively. They reveal that a piezoelectric response range of 3.9–5.6 nm is acquired from TCN, comparable to that reported for carbon nitride.^52^ The 2D topological graph and the corresponding 2D and 3D piezoresponse amplitude images of 10-MoS_2_/TCN are shown in Figure 3d–f, respectively. Certain regions of 10-MoS_2_/TCN exhibit piezoelectric responses exceeding 10 nm, significantly surpassing those of TCN under the same applied electric field. Compared to the SEM-EDS mapping images shown in Figure S2, the regions of high piezoelectric response observed in Figure 3d–f correspond to the MoS_2_ nanosheets. Accordingly, the strong piezoelectrical characteristic of 10-MoS_2_/TCN originates from the MoS_2_ nanosheets on carbon nitrides.

**Figure 4:**
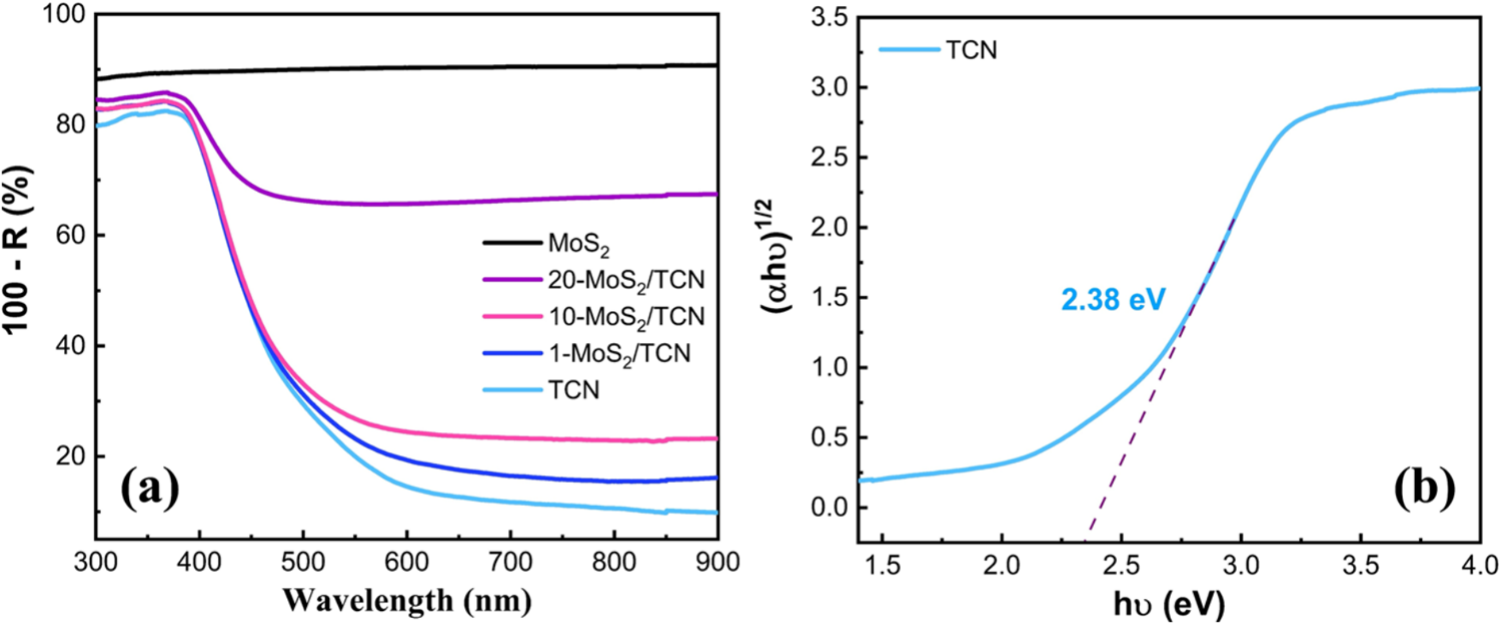
(a) UV–vis DRS of TCN, MoS_2_, and MoS_2_/TCN heterostructures. (b) Tauc plot of TCN.

Figure 4a shows the UV–vis diffuse reflectance spectra (DRS) of the TCN, MoS_2_, and MoS_2_/TCN heterostructures. The absorption edge at 520 nm is observed from the DRS of TCN. The corresponding band gap of 2.38 eV is determined by the Tauc plot illustrated in Figure 4b. Compared to conventional carbon nitrides,^41^ the light-harvesting ability of TCN significantly extends toward the visible light range. With the MoS_2_ nanosheets on the surface, the MoS_2_/TCN heterostructures exhibit the same absorption edge as TCN, as displayed in Figure 4a. Compared to the spectra of TCN and MoS_2_, the absorptions of MoS_2_/TCN heterostructures at λ > 520 nm increasing with the MoS_2_ loading amount are attributed to the deposition of MoS_2_ nanosheets on TCN.

### 3.2 Piezophotocatalytic HER over TCN and MoS_2_/TCN Heterostructures

**Figure 5:**
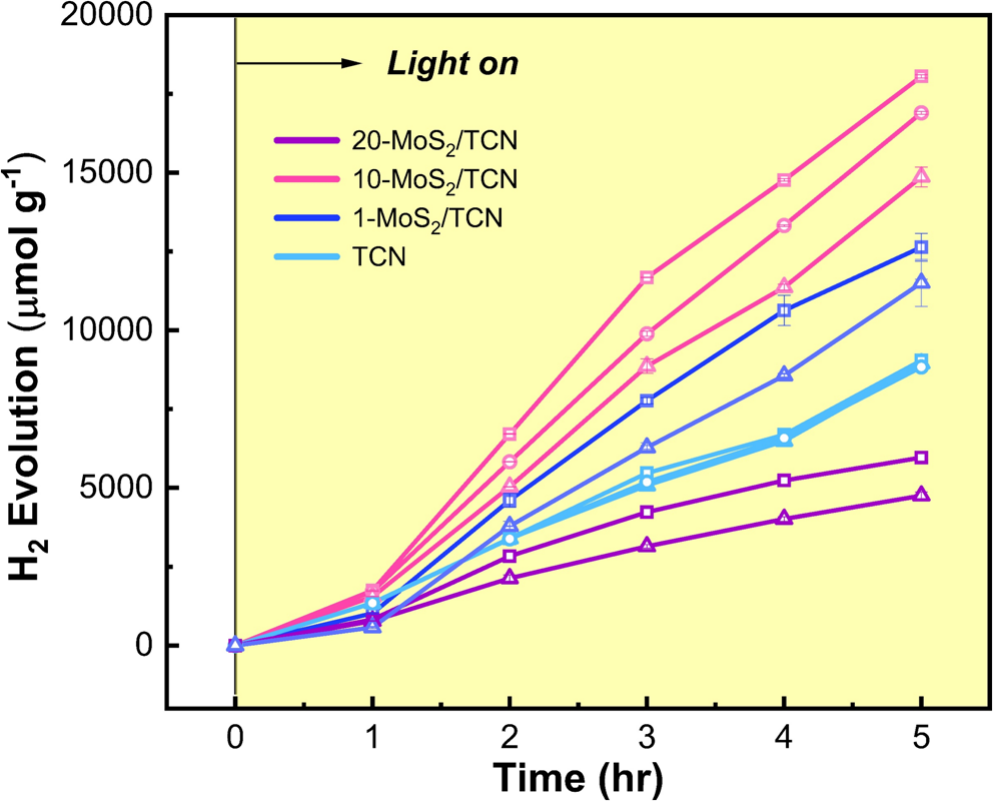
Piezophotocatalytic H_2_ evolution over TCN and MoS_2_/TCNs under visible light irradiation and varying vortex speed (triangle: 500, circle: 900, square: 1300 rpm). The error bars represent the standard deviations calculated from three replicates.

The piezophotocatalytic hydrogen evolution reactions (HER) over MoS_2_/TCN heterostructures were conducted in a customized quartz reactor under AM 1.5G (100 mW cm^–2^) irradiation through a 420 nm cutoff filter coupled to various vortex speeds. Figure 5 shows the amount of H_2_ evolution over 1-MoS_2_/TCN, 10-MoS_2_/TCN, and 20-MoS_2_/TCN under vortex speeds of 500, 900, and 1300 rpm. In these reactions, 3 wt % Pt and 10 vol % TEOA were used as the cocatalyst and hole scavenger, respectively. For comparison, photocatalytic HER over TCN under the three vortex speeds was also examined in this work. H_2_ yields over TCN at all speeds are almost identical to 1.8 mmol g^–1^ h^–1^, as shown in Figure 5, indicating a lack of induced polarization in TCN by shearing force from the vortex fluid motion. Moreover, the same HER rates over TCN indicate that mass transfer resistance does not play a significant role in the catalytic reactions under vortex speeds of 500–1300 rpm.

In contrast, the boosted HER rates acquired under high-speed vortex fluid motion for each MoS_2_/TCN heterostructure are presented in Figure 5. Rather than being influenced by mass transfer, the higher HER rate observed at increased vortex speeds for all MoS_2_/TCN heterostructures is attributed to stronger piezoelectric polarization induced by the more intense shear forces at higher vortex speeds. This conclusion is supported by the piezoelectric response measurements of TCN and 10-MoS_2_/TCN shown in Figure 3, where the piezoelectric response of 10-MoS_2_/TCN is significantly greater than that of TCN under the same applied electric field. Compared to that of TCN, the enhanced HER rate in MoS_2_/TCN heterostructures clearly stems from the deposited MoS_2_ nanosheets, which amplify the piezoelectric effect. Under the same vortex speed, the HER rate over 1-MoS_2_/TCN and 10-MoS_2_/TCN is superior to that of TCN. However, upon further increasing the MoS_2_ loading on carbon nitrides, the HER rate of 20-MoS_2_/TCN is significantly inferior to TCN. HER rate over the four catalysts increases in the order 20-MoS_2_/TCN < TCN < 1-MoS_2_/TCN < 10-MoS_2_/TCN. 10-MoS_2_/TCN exhibits the highest HER activity of 3.62 mmol g^–1^ h^–1^ under visible-light illumination coupled with fluid mechanical energy, which is 2-fold enriched compared to TCN. Poor HER activity of 20-MoS_2_/TCN is likely ascribed to the higher concentration of MoS_2_ nanosheets on carbon nitride, which may reduce photon absorption by the underlying carbon nitrides.^40^ This also suggests that rather than those generated in the MoS_2_ portion, photogenerated charges in the carbon-rich carbon nitride portion of MoS_2_/TCN heterostructures with appropriate potentials play a critical role in the surface redox reactions. The details are discussed in Section 3.4.

Figure S4a,b shows the high-angle annular dark-field (HAADF) STEM and TEM images of 10-MoS_2_/TCN after piezophotocatalytic HER, revealing that 10-MoS_2_/TCN keeps the morphology of 2D curly sheets after HER. The HAADF STEM image in Figure S4a displays that Pt nanoparticles are well distributed on 10-MoS_2_/TCN. Figure S4c,d shows the HRTEM images of the portion denoted in Figure S4b. The lattice spacings exhibited in Figure S4c,d correspond to the *d*-spacings of MoS_2_ (002) and C_3_N_4_ (002), respectively, indicating that the crystal structures of MoS_2_ and TCN in 10-MoS_2_/TCN are preserved after 5 h HER.

### 3.3 Piezophotocatalytic Tetracycline Degradation over TCN and MoS_2_/TCN Heterostructures

**Figure 6:**
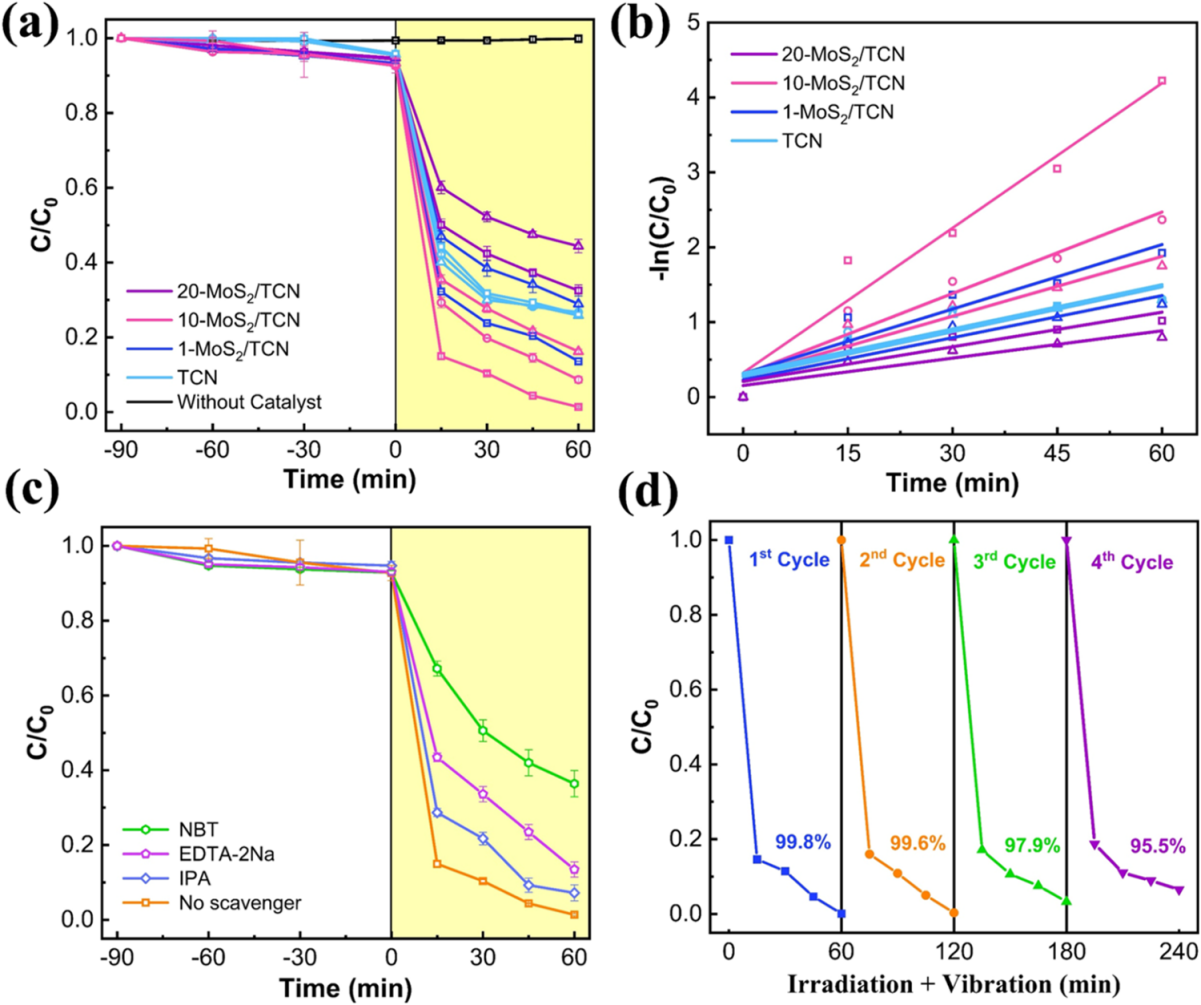
(a) Piezophotocatalytic TC degradation using TCN and MoS_2_/TCNs under visible-light irradiation and varying vortex speed (triangle: 500, circle: 900, square: 1300 rpm). (b) First-order kinetic model of the TC degradation reaction (triangle: 500, circle: 900, square: 1300 rpm). (c) Piezophotocatalytic TC degradation using 10-MoS_2_/TCN with sacrificial agents under visible-light irradiation and vortex speed at 1300 rpm. (d) Stability of 10-MoS_2_/TCN for piezophotocatalytic TC degradation under visible-light irradiation and vortex speed at 1300 rpm. The error bars represent the standard deviations calculated from three replicates.

In addition to HER, piezophotocatalytic tetracycline (TC) degradation using MoS_2_/TCN heterostructures was also carried out in this work. It has been reported that direct photodegradation of TC aqueous solution occurs under UV irradiation.^53,54^ To carefully examine the piezophotocatalytic activity of MoS_2_/TCN heterostructures for TC degradation, the piezophotocatalytic reactions were carried out under AM 1.5G (100 mW cm^–2^) irradiation through a 420 nm cutoff filter coupled to various vortex speeds. Unlike HER, TC degradation was conducted in an aerobic condition with no cocatalyst and hole scavenger in the TC aqueous solution. Figure 6a displays the piezophotocatalytic performances of TCN and MoS_2_/TCN heterostructures for TC degradation, showing the normalized TC concentration (*C*/*C*_0_) as a function of time. In the absence of a catalyst, TC is not degraded under visible light irradiation coupled with a vortex speed of 1300 rpm. With the addition of catalysts to the solution, the adsorption of TC on catalysts is observed from the decrease in normalized concentration measured under various vortex speeds in the dark for 90 min. The TC normalized concentration remains constant using every catalyst, while further prolonging the period in the dark. Accordingly, the degradation of TC was then examined by turning on visible-light irradiation after 90 min in the dark.

Figure 6a shows a trend similar to that of the HER rate: the TC degradation rate over TCN remains nearly unaffected by the vortex speed. After 60 min of reactions over TCN, the normalized TC concentrations decrease to 26.8% across all three vortex speeds of 500, 900, and 1300 rpm. Nevertheless, the degradation of TC over each MoS_2_/TCN heterostructure is enhanced by speeding up the vortex fluid motion, confirming the fluid-mechanical induced piezoelectric boost to the photocatalytic performance of MoS_2_/TCN heterostructures. Due to the low concentration of photogenerated charges in the carbon nitride portion, 20-MoS_2_/TCN exhibits poorer catalytic performance for TC degradation compared to TCN. At a vortex speed of 1300 rpm, the TC photocatalytic degradation rate of the catalysts follows the order: 20-MoS_2_/TCN < TCN < 1-MoS_2_/TCN < 10-MoS_2_/TCN. Among the three MoS_2_/TCN heterostructures, 10-MoS_2_/TCN shows the highest TC degradation activity. After a 60 min piezophotocatalytic reaction over 10-MoS_2_/TCN, 83.9, 91.3, and 98.8% of TC in the solutions are degraded at vortex speeds of 500, 900, and 1300 rpm, respectively. Figure S5 presents the absorption spectra of the TC solution during the piezophotocatalytic reaction using 10-MoS_2_/TCN. The characteristic absorption peaks of TC at 357 and 275 nm, attributed to the amide group on the aromatic ring and the aromatic ring with a phenolic hydroxyl group, respectively,^55^ nearly vanish after 60 min of piezophotocatalysis with 10-MoS_2_/TCN. This indicates the fracture of the TC structure during the piezophotocatalytic process.

Figure 6b reveals that the catalytic TC degradation reactions over TCN and MoS_2_/TCN heterostructures follow the first-order reaction kinetics.^56^ The calculated rate constants (*k*, min^–1^) are listed in Table 1. As shown in Figure 6a, the blank experiment demonstrates that TC cannot be directly degraded under visible light irradiation. Therefore, the rate constants presented in Table 1 are based solely on the piezophotocatalytic degradation of TC over TCN and MoS_2_/TCN heterostructures. The rate constants of MoS_2_/TCN heterostructures increase with the vortex speed, indicating that the shearing force on MoS_2_/TCN heterostructures boosts the photocatalytic kinetics for TC degradation. In comparison with the rate constant of 0.020 min^–1^ for TCN, 10-MoS_2_/TCN achieves a rate constant of 0.064 min^–1^ at 1300 rpm, which is the highest one among all conditions, attributed to the optimized photo- and piezo coupling effect.

**Table 1:** Reaction Rate Constants (min^–1^) for TC Degradation over TCN and MoS_2_/TCN Heterostructures under Visible-Light Illumination Coupled to Various Vortex Speeds

catalyst	500 rpm	900 rpm	1300 rpm
TCN	0.020	0.020	0.020
1-MoS_2_/TCN	0.018	-	0.029
10-MoS_2_/TCN	0.027	0.036	0.064
20-MoS_2_/TCN	0.012	-	0.015

The oxidation mechanism of piezophotocatalytic TC degradation over 10-MoS_2_/TCN was further investigated by adding different sacrificial agents for reactive oxygen species (ROS) and holes in TC aqueous solutions during the reaction.^57^ Nitroblue tetrazolium chloride (NBT), isopropyl alcohol (IPA), and ethylenediaminetetraacetic acid disodium (EDTA-2Na) are employed to quench superoxide radicals (^·^O_2_^–^), hydroxyl radicals (OH^·^), and holes during piezophotocatalytic TC degradation, respectively. As shown in Figure 6c, the TC degradation rate over 10-MoS_2_/TCN is significantly reduced by respectively adding NBT and EDTA-2Na to the solution compared to the one without adding any sacrificial agent. These results reveal that superoxide radicals and holes are the major active species for piezophotocatalytic TC degradation over 10-MoS_2_/TCN. The stability of 10-MoS_2_/TCN for piezophotocatalytic TC degradation was further examined under visible-light illumination coupled with a vortex speed at 1300 rpm for four sequential cycles. Figure 6d shows that 10-MoS_2_/TCN maintains a superior stable piezophotocatalytic TC degradation activity after four cycles. The slightly reduced performance results from ∼2 wt % loss of the catalyst during the lyophilization process after each cycle.

As summarized in Table S1, the MoS_2_/TCN heterostructure exhibits promising performance in piezophotocatalytic TC degradation compared to contemporary studies.^31−33,52^ The TC removal efficiency achieved with MoS_2_/TCN, driven by sustainable fluid mechanical energy and visible light, is comparable to that of reported piezophotocatalytic systems that utilize simulated sunlight (including UV) combined with either ultrasonication^32,33^ or high-speed stirring.^52^ This study highlights an energy-efficient approach to environmental remediation, where the shearing forces generated by fluid flow, essential for wastewater discharge, piezoelectrically enhance the visible-light-driven photocatalytic efficiency of the MoS_2_/TCN heterostructure. However, like other nanomaterials, MoS_2_-based catalysts pose potential risks of harmful interactions with the eco-environment during contaminant removal.^58^ A thorough understanding of these interactions with biological systems is essential for the development of safe and sustainable green technologies.

### 3.4 The Function of MoS_2_ Nanosheet and the Principle of Piezophotocatalytic Reaction over MoS_2_/TCN Heterostructure

The Mott–Schottky plots and XPS valence band spectra of TCN and 10-MoS_2_/TCN are shown in Figures S6 and S7, respectively. Combining the results shown in Figures 4, S6, and S7, the band diagrams of TCN and 10-MoS_2_/TCN are illustrated in Figure S8,^59^ where the conduction and valence band-edge potentials with respect to the reversible hydrogen electrode (RHE) have been determined (Supporting Information). It shows that the conduction band edges of TCN and 10-MoS_2_/TCN are more negative than the formation potentials of H_2_ and ^•^O_2_^–^, while the valence band edges are located more positively than the oxidation potential of TEOA. Accordingly, both TCN and 10-MoS_2_/TCN are thermodynamically capable of the photocatalytic HER with the assistance of TEOA and the photocatalytic TC degradation through ^•^O_2_^–^.

**Figure 7:**
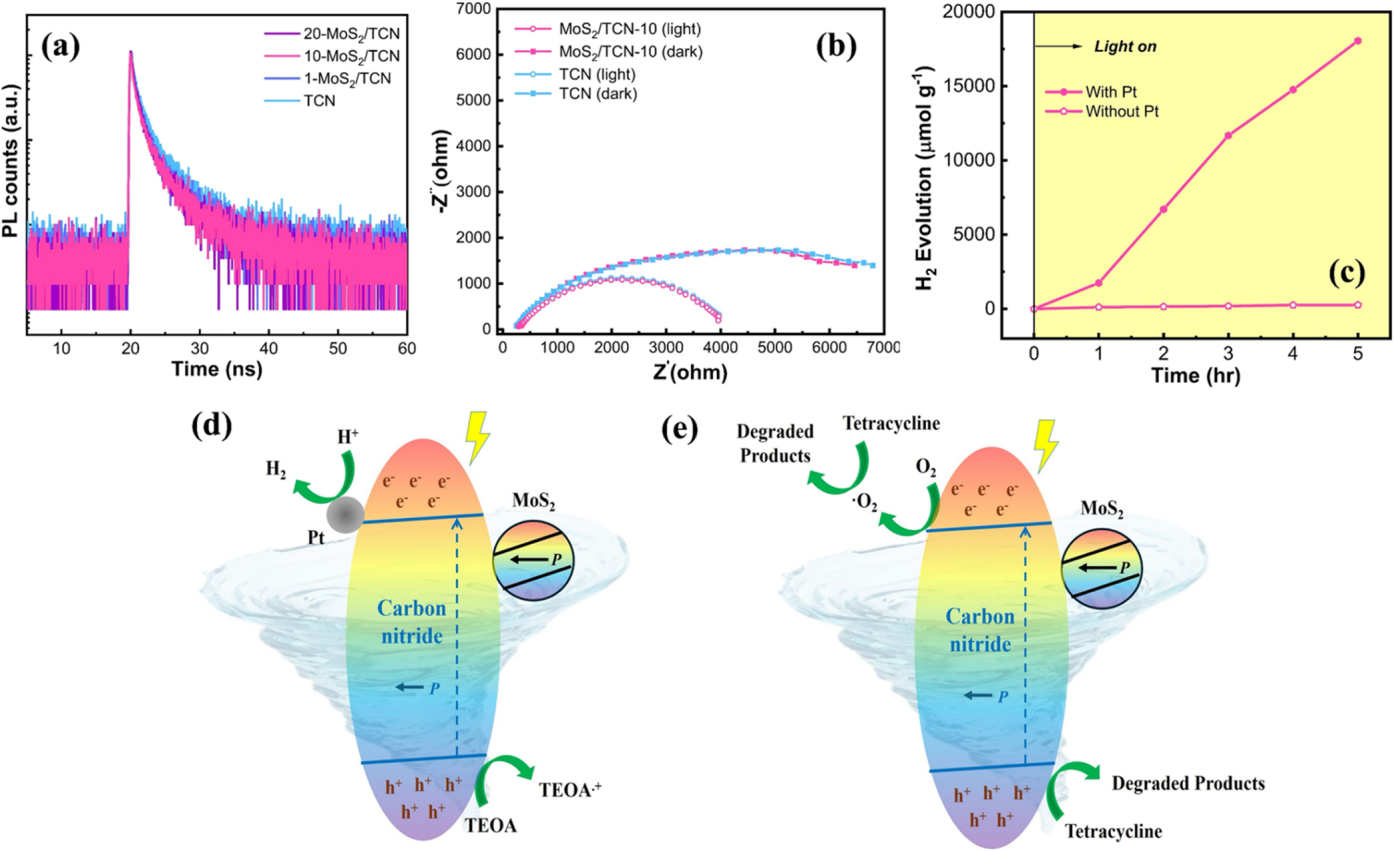
(a) TRPL decay curves of TCN and 10-MoS_2_/TCN. (b) Nyquist plots of TCN and 10-MoS_2_/TCN measured in the dark and under illumination. (c) Piezophotocatalytic HER over 10-MoS_2_/TCN with and without Pt cocatalyst. Proposed principles for (d) piezophotocatalytic HER with the assistance of TEOA and (e) piezophotocatalytic TC degradation over 10-MoS_2_/TCN under visible light coupled to a vortex flow.

As shown in Figures 5 and 6a, the photocatalytic activities of MoS_2_/TCN heterostructures are boosted by accelerating the vortex speed, whereas the vortex speed does not influence that of TCN. It is concluded that the piezoelectric characteristics of MoS_2_/TCN heterostructures originate from the deposited MoS_2_ nanosheets. In addition to being the piezopotential generator of the heterostructures, the role of MoS_2_ nanosheets in the photocatalytic activity of MoS_2_/TCN heterostructures was further inspected in this work. The dynamic of band-edge recombination in TCN and 10-MoS_2_/TCN was investigated by using time-resolved photoluminescence (TRPL) in the absence of shearing force. The TRPL decay curves of TCN and 10-MoS_2_/TCN at 520 nm are shown in Figure 7a. The average PL lifetimes of both TCN and 10-MoS_2_/TCN are 1.6 ns. The identical PL lifetimes of TCN and 10-MoS_2_/TCN, acquired without applying shearing force, indicate no charge transfer from carbon nitride to MoS_2_ in 10-MoS_2_/TCN.

The charge transfer characteristics from TCN and 10-MoS_2_/TCN to the electrolyte solution were also investigated by using electrochemical impedance spectroscopy (EIS). Figure 7b displays the Nyquist plots of TCN and 10-MoS_2_/TCN measured in the dark and under illumination. Identical semicircular arcs are acquired from TCN and 10-MoS_2_/TCN both in the dark and under illumination. The EIS results reveal that the charge transfers from these two catalysts to the electrolyte solutions experience the same pathway where the active sites of TCN and 10-MoS_2_/TCN are located on the carbon-rich carbon nitrides. Moreover, as shown in Figure 7c, no H_2_ is produced over 10-MoS_2_/TCN in the absence of cocatalyst Pt under irradiation coupled with a vortex speed of 1300 rpm. This result reflects that MoS_2_ nanosheets do not function as cocatalysts in the piezophotocatalytic reactions.

In the case of 10-MoS_2_/TCN, the deposited MoS_2_ nanosheets on carbon-rich carbon nitrides do not contribute to significant light absorption to limit the generation of photocharged carriers in the carbon-rich carbon nitride matrix, as shown in Figure 4a. The results of PFM measurements and piezophotocatalytic examinations, respectively shown in Figures 3, 5, and 6a, indicate that the strong piezoelectrical characteristic of 10-MoS_2_/TCN originates from the MoS_2_ nanosheets on carbon-rich carbon nitrides. On the other hand, the results displayed in Figure 7a–c provide solid evidence that no charge transfer occurs between MoS_2_ and carbon nitride, and MoS_2_ nanosheets do not function as cocatalysts in the piezophotocatalytic reactions. Figure 7d,e illustrates the proposed principles for piezophotocatalytic HER with the assistance of TEOA and piezophotocatalytic TC degradation using 10-MoS_2_/TCN under visible light coupled with vortex flow, respectively. MoS_2_ nanosheets as a piezoelectric generator, triggered by fluid mechanical energy, induce significant in-plane piezoelectric potential in 2D carbon-rich carbon nitride. The induced macroscopic piezoelectric potential combined with the visible-light-harvesting ability of carbon-rich carbon nitride facilitates the collection of photogenerated electrons and holes on the carbon nitride surface to boost the HER and TC degradation activities over the MoS_2_/TCN heterostructure. Accordingly, in addition to efficiently producing green hydrogen, 10-MoS_2_/TCN—responsive to both visible light and fluid mechanical energy—enables the piezophotocatalytic degradation of organic pollutants in wastewater discharge by harnessing solar and fluid mechanical energies available in nature.

## 4 Conclusions

In this study, MoS_2_/TCN heterostructures are constructed by solvothermal growth of MoS_2_ nanosheets on carbon-rich carbon nitrides. The MoS_2_/TCN heterostructures, which are sensitive to both visible light and fluid mechanical energy, are developed by coupling the visible-light photocatalytic properties of carbon-rich carbon nitride with the piezoelectric properties of MoS_2_ for piezophotocatalytic HER and TC degradation. MoS_2_/TCN heterostructure exhibits the same absorption edge as TCN at 520 nm, which is significantly red-shifted compared to conventional carbon nitride. The deposited MoS_2_ nanosheets on TCN contribute to the increased absorptions of MoS_2_/TCN heterostructures at λ > 520 nm with the MoS_2_ loading amount. As characterized by piezoelectric force microscopy, the piezopotential response from the MoS_2_/TCN heterostructure is much greater than that from TCN in response to the same applied voltage. Compared to TCN, the photocatalytic performance of MoS_2_/TCN heterostructures for HER and TC degradation under visible light irradiation and the same vortex speed is improved by increasing the MoS_2_ loading to an optimal amount. The poor activity of MoS_2_/TCN heterostructure with high MoS_2_ loading is likely ascribed to the reduction of photon absorption of the carbon nitrides by the overdeposited MoS_2_ nanosheets. The boosted HER and TC degradation rates are acquired under high-speed vortex-induced fluid motion for each MoS_2_/TCN heterostructure, whereas HER and TC degradation rates over TCN at all speeds are almost identical. Compared to TCN, the piezophotocatalytic activities of optimized MoS_2_/TCN heterostructure increase the HER rate from 1.8 to 3.62 mmol g^–1^ h^–1^ and the rate constant for TC degradation from 0.020 to 0.064 min^–1^, respectively. The shearing force induced by the vortex fluid motion, necessary for well-dispersed catalysts in solution, piezoelectrically boosts the photocatalytic activity of the MoS_2_/TCN heterostructure. TRPL and EIS measurements confirm that no charge transfer occurs between MoS_2_ and TCN. Accordingly, the MoS_2_ nanosheet functions as the piezopotential generator of the heterostructures to activate the piezopotential in carbon nitrides but not as a cocatalyst for piezophotocatalytic reactions. The induced macroscopic piezoelectric potential combined with the visible-light-harvesting ability of carbon-rich carbon nitride facilitates the collection of photogenerated electrons and holes on the carbon nitride surface to boost the HER and TC degradation activities of the MoS_2_/TCN heterostructure.

## Supporting Information Available

SEM-EDS mapping images, N_2_ adsorption–desorption isotherms, Mott–Schottky plots, XPS valence band spectra, and band diagrams of TCN and 10-MoS_2_/TCN; TEM characterization of 10-MoS_2_/TCN after HER; and absorption spectra of the TC solution during the piezophotocatalytic reaction (PDF)
