# Supplementary material for: Fluid Mechanical and Visible-Light-Driven Piezophotocatalysis in MoS2/Carbon-Rich Carbon Nitride Heterostructures for Enhanced Green Energy Production and Environmental Remediation
Source: ACS Appl Mater Interfaces. 2025 Mar 1;17(10):15544–54. doi: 10.1021/acsami.5c01107 (PMC11912202; doi:10.1021/acsami.5c01107)
Supplement: Supplementary file 1 — am5c01107_si_001.pdf [file am5c01107_si_001.pdf]

Supporting Information

Fluid-Mechanical and Visible-Light-Driven Piezophotocatalysis in MoS_2/Carbon-Rich Carbon Nitride Heterostructures for Enhanced Green Energy Production and Environmental Remediation

Chien-Jung Wu‡, Sin-Cin He‡, Tzu-Chi Kuo, Jih-Jen Wu*

Department of Chemical Engineering, National Cheng Kung University, Tainan 701, Taiwan

‡These two authors contributed equally to this work

*Corresponding Author. Email: wujj@mail.ncku.edu.tw

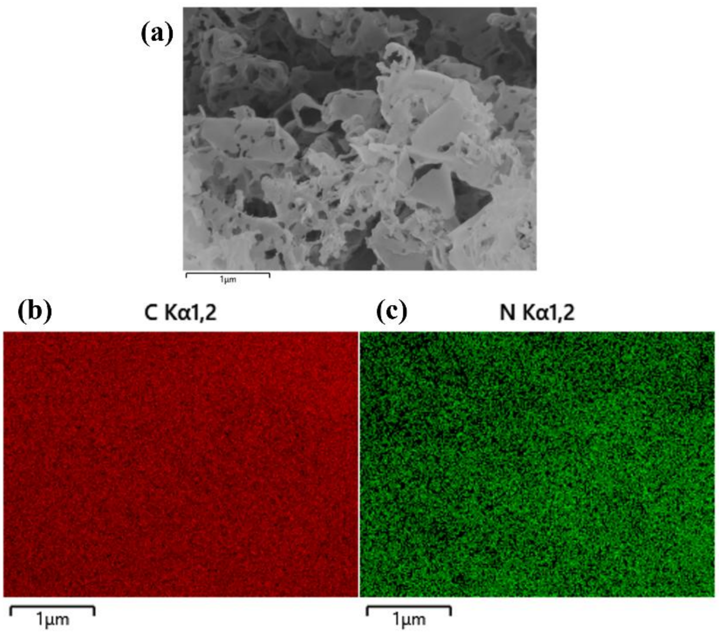

Figure S1. (a) SEM image of TCN and the corresponding elemental SEM-EDS mapping images of (b) C and (c) N.

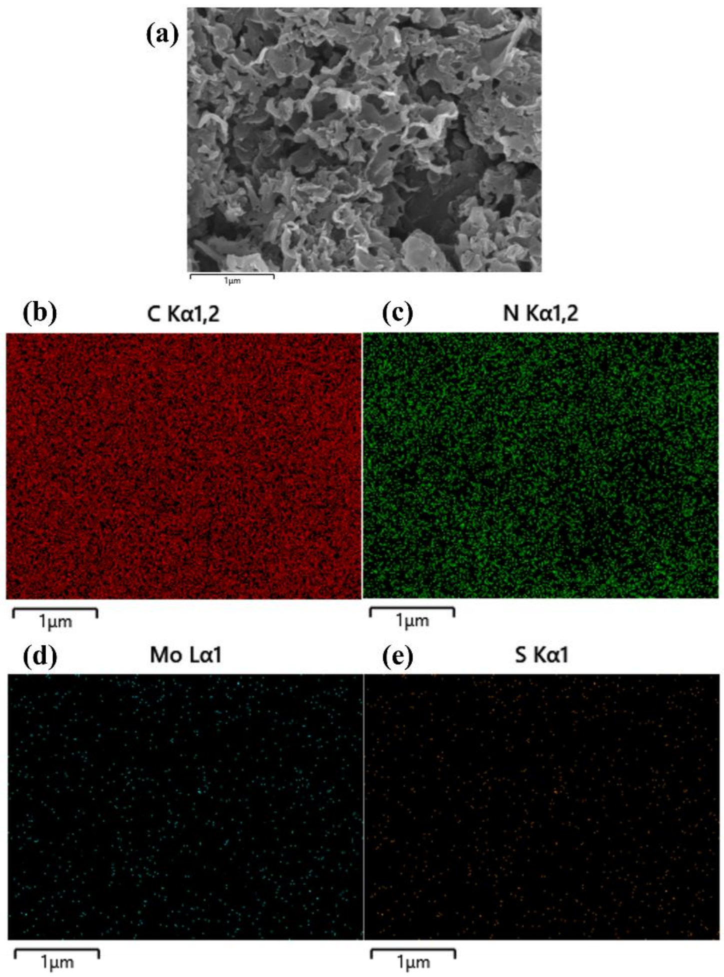

Figure S2. (a) SEM image of 10-MoS_2/TCN and the corresponding elemental SEM-EDS mapping images of (b) C, (c) N, (d) Mo, and (e) S.

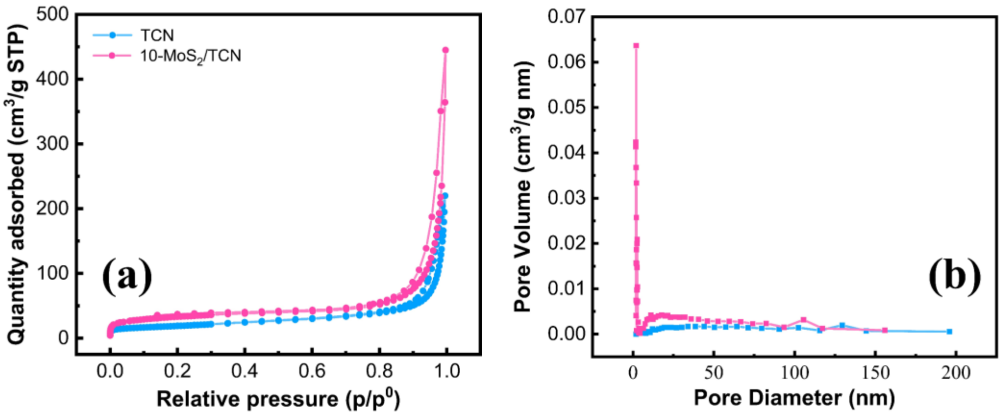

Figure S3. (a) N_2 adsorption-desorption isotherms and (b) pore size distributions of TCN and 10-MoS_2/TCN.

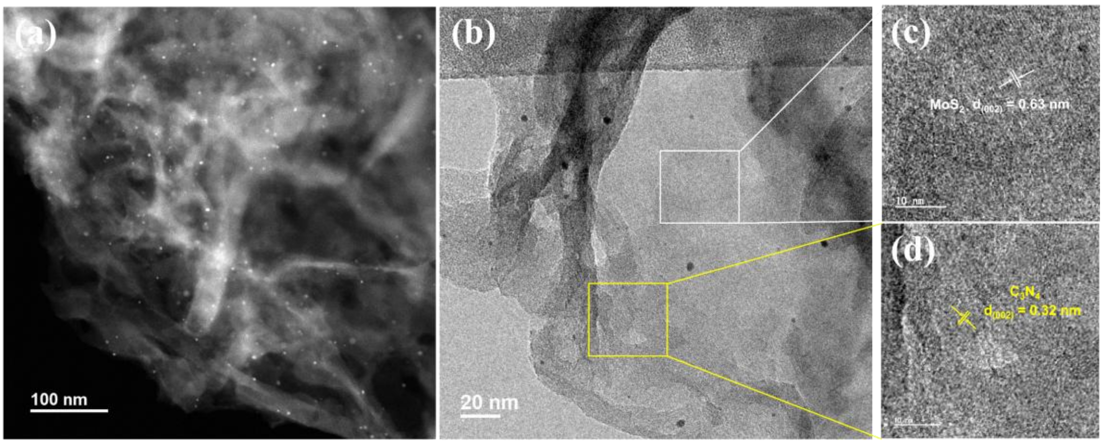

Figure S4. (a) HAADF STEM image of 10-MoS_2/TCN after HER. (b) TEM image of 10-MoS_2/TCN after HER. (c) and (d) HRTEM images of the portions denoted in (b).

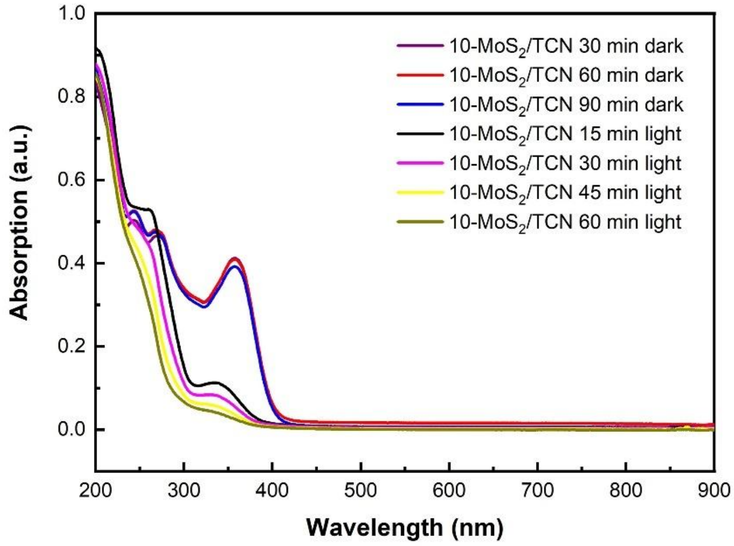

Figure S5. Absorption spectra of the TC solution during the piezophotocatalytic reaction using 10-MoS_2/TCN.

Table S1. Recently reported works for piezophotocatalytic TC degradation.

Catalyst	Light source	Source of mechanical force	TC concentration (mg L^{-1})	k (min^{-1})	Removal efficiency (%) /Time (min)	Ref.
$Zn_{(1-x)}Ce_xO$	Visible light	Ultrasonication	50	--	82 / 150	1
CdZnS/BiOCl	Simulated sunlight (300W)	Ultrasonication (80W, 40kHz)	20	0.184	99.0 / 30	2
$NaNbO_3$/g-C_3N_4	Simulated sunlight (300W)	High-speed stirring (1000 rpm)	10	0.176	87.3 / 10	3
Carbon quantum dots/$BiVO_4$	Simulated sunlight (300 W)	Ultrasonication (100W)	10 / 30	0.052	~70 / 30	4
MoS_2/TCN	Visible light (100 mW cm^{-2})	Low-frequency vortex (1300 rpm)	10	0.064	85.1 / 15<br>98.8 / 60	*This work*

References

(1) Elmehdi, H. M.; Ramachandran, K.; Chidambaram, S.; Mani, G. T.; Pandiaraj, S.; Alqarni, S. A.; Daoudi, K.; Gaidi, M. Diode Characteristics, Piezo-Photocatalytic Antibiotic Degradation and Hydrogen Production of Ce^{3+} Doped ZnO Nanostructures. *Chemosphere* **2024**, *350*, 141015.

(2) Zhang, J.; Sun, X.; Zhu, W.; Liu, G.; Xian, T.; Yang, H. Design of CdZnS/BiOCl Heterostructure as a Highly-Efficient Piezo-Photocatalyst for Removal of Antibiotic. *J. Environ. Chem. Eng.* **2024**, *12*, 114405.

(3) Guo, L.; Hu, C.; Tu, S.; Wang, C.; Mei, L.; Zhang, Y.; Huang, H. Weak Force-Polarization Driven Exceptional Piezo-Photocatalysis by Coupling Dual-Active Piezoelectric Semiconductors in $NaNbO_3$/g-C_3N_4 Heterojunction. *Chem. Eng. J.* **2023**, *476*, 146541.

(4) Lv, M.; Wang, S.; Shi, H. Carbon Quantum Dots/$BiVO_4$ S-Scheme Piezo-Photocatalysts Improved Carrier Separation for Efficient Antibiotic Removal. *J. Mater. Sci. Technol.* **2024**, *201*, 21–31.

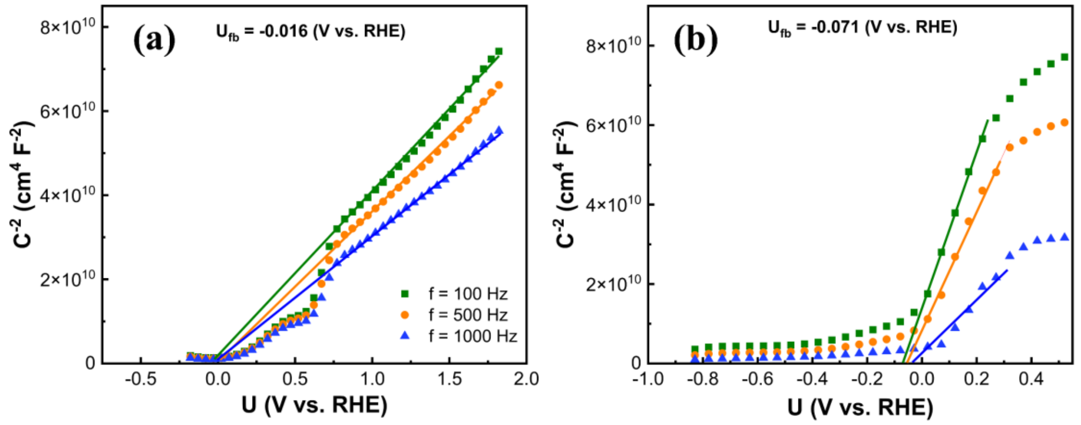

Figure S6. Mott-Schottky plots of (a) TCN and (b) 10-MoS_2/TCN.

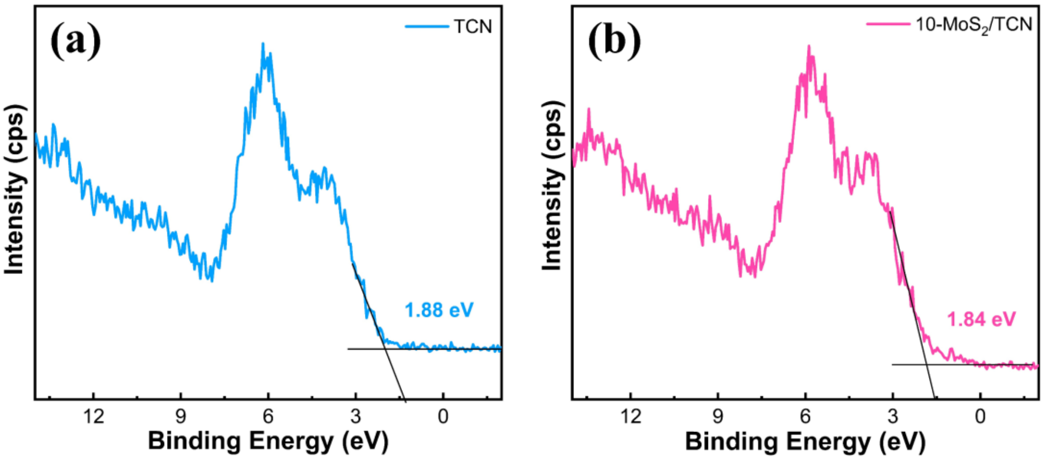

Figure S7. XPS-valance band spectra of (a) TCN and (b) 10-MoS_2/TCN.

Construction of band diagrams of TCN and 10-MoS_2/TCN

The electronic band edge positions for TCN and 10-MoS_2/TCN shown in Figure S8 are determined by combining the results of the UV-vis diffuse reflectance spectra (Figure 4), the Mott-Schottky plots (Figure S6), and the XPS-valance band (VB) spectra (Figure S7).[1-3] The flat band potential for TCN and 10-MoS_2/TCN, which is derived by extrapolating the curve to the x-intercept in the Mott–Schottky plot, represents the corresponding Fermi level (E_F) vs. RHE.[4] The energy difference between the Fermi level and the valance band maximum (VBM), i.e. E_F-E_{vmax}, is obtained by extrapolating a linear fit to the leading edge of the XPS-VB spectra to the baseline.[5] The valence band edge positions of TCN and 10-MoS_2/TCN are determined based on the results of the XPS-VB spectra and the Mott-Schottky plots. The conduction band edge positions are further derived using the band gap values acquired from UV-vis diffuse reflectance spectra.

References:

1. Wu, P.-S.; Lin, T.-J.; Hou, S.-S.; Chen, C.-C.; Tsai, D.-L.; Huang, K.-H.; Wu, J.-J. Non-Photochromic Solar Energy Storage in Carbon Nitride Surpassing Blue Radicals for Hydrogen Production. *J. Mater. Chem. A* **2022**, *10*, 7728–7738. https://doi.org/10.1039/D1TA10091B.
2. Chen, C.-C.; Tsai, D.-L.; Liu, H.-T.; Wu, J.-J. Carbon Vacancy-Modified Carbon Nitride Allotropic Composite for Solar Hydrogen Generation Coupled with Selective Oxidation of 5-Hydroxymethylfurfural. *ACS Sustainable Chem. Eng.* **2023**, *11*, 6435–6444. https://doi.org/10.1021/acssuschemeng.3c00363.
3. Chueh, L.-C.; Lin, T.-J.; Lee, H.-C.; Wu, J.-J. Defective Potassium Poly(Heptazine Imide) Preventing Spin Delocalization and Hole Transfer Deactivation for Efficient Solar Energy Conversion and Storage. *Small* **2024**, *20*, 2304813. https://doi.org/10.1002/smll.202304813.
4. Zhang, Z.; Li, B.; Qian, Q.; Tang, X.; Hua, M.; Huang, B.; Chen, K. J. Revealing the Nitridation Effects on GaN Surface by First-Principles Calculation and X-Ray/Ultraviolet Photoemission Spectroscopy. *IEEE Trans. Electron Dev.* **2017**, *64*, 4036-4043. https://doi.org/10.1109/TED.2017.2733547.
5. Hankin, A.; Bedoya-Lora, F. E.; Alexander, J. C.; Regoutz, A.; Kelsall, G. H. Flat Band Potential Determination: Avoiding the Pitfalls. *J. Mater. Chem. A* **2019**, *77*, 26162-2617. https://doi.org/10.1039/C9TA09569A.

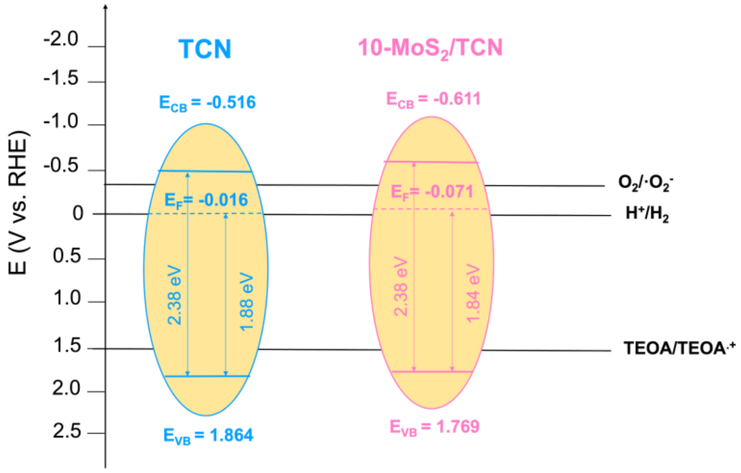

Figure S8. Band diagrams of TCN and 10-MoS_2/TCN.
